# Health status of regularly physically active persons with spinal cord injury

**DOI:** 10.1038/s41394-017-0033-8

**Published:** 2017-12-22

**Authors:** Lene C. Vik, Anne M. Lannem, Britt Marie Rak, Trine Stensrud

**Affiliations:** 1 Norwegian School of Sport Sciences, Oslo, Norway; 2 Sunnaas Rehabilitation Hospital, Nesodden, Norway

## Abstract

**Study design:**

A non-controlled cross-sectional study.

**Objectives:**

To make a descriptive examination of health status in persons with paraplegia and tetraplegia who exercise regularly according to Canadian guidelines.

**Settings:**

Sunnaas Rehabilitation Hospital and the Norwegian School of Sport Sciences.

**Methods:**

Eighteen persons (men/women = 9/9), aged 41‒72 years with spinal cord injury (SCI), who exercise regularly were included. Post-injury years ranged from 4 to 48 years. Clinical examination of body composition, bone mineral density (BMD), forced vital capacity (FVC), forced expiratory volume in one second (FEV_1_), diffusion capacity (DL_CO_), cardiorespiratory fitness (VO_2max_), and self-reported quality of life (QOL) obtained by questionnaire was performed. Lung function results are presented as % predicted and VO_2max_ as absolute values relative to body weight. All results are given as median and range.

**Results:**

Persons with paraplegia (*n* = 13) were defined as overweight with fat mass 42% (25‒51). BMD 1.047 g cm^−2^ (0.885‒1.312) was within normal range. FVC 95% predicted (60‒131), FEV_1_ 90% predicted (61‒119), DL_CO_ 77% predicted (56‒103), and VO_2max_ 16.66 ml kg^−1^ min^−1^ (12.15‒25.28) defined good aerobic capacity according to age controlled reference values (18). Persons with tetraplegia (*n* = 5) were slightly overweight with fat mass 35% (26‒47). BMD 1.122 g cm^−2^ (1.095‒1.299) was within normal range. FVC 72% predicted (46‒91), FEV_1_ 75% predicted (43‒83), DL_CO_ 67% predicted (56‒84), and VO_2max_ 16.70 ml kg^−1^ min^−1^ (9.91‒21.01) defined excellent aerobic capacity according to reference values (18). QOL was ranked as median 7.5 (0‒10 scale).

**Conclusions:**

Persons with SCI who exercise regularly following the Canadian guidelines responded with rather positive associations for health outcomes. Additional research is needed to strengthen our findings.

## Introduction

Persons with spinal cord injuries (SCI) are reported to be especially inclined to develop secondary health complications. It has been suggested that these complications are responsible for the increased mortality in persons with chronic SCI and are related to potentially treatable factors. Osteoporosis, type II diabetes, and heart disease are examples of conditions that are related to inactivity [1]. However, depending on the level and completeness of injury, SCI may cause varying extents of disability. For some persons, this will limit their ability to perform physical activity in certain ways and can explain the most common cause of deconditioning in this population. It has also been suggested that loss of independence and physical fitness may lead to withdrawal from society and may negatively impact the quality of life (QOL) [2].

Positive associations between health and physical activity in the SCI population have been well established [3]. Even so, the level of physical activity varies. Jørgensen and coauthors investigated participation in leisure time physical activity (LTPA) among older adults with long-standing SCI [4]. Almost one-third reported no LTPA during a day. Of those who did, walking and wheeling were the most prevalent activities. Half of the participants performed less than five minutes LTPA at moderate to vigorous intensity [4]. To gain health benefits, activities at this intensity level are required [5]. To improve health in terms of fitness and QOL, promoting physical activity is considered to be of great importance. In addition, Canadian researchers have developed evidence-informed, consensus physical activity guidelines targeting persons with SCI [5]. These guidelines indicate type, amount, and intensity of physical activity. An evaluation showed improvements in certain aspects of aerobic and muscular fitness [6]. However, whether the guidelines are robust enough to provide health benefits is discussed [7‒9].

It has been reported that there is a lack of health programs promoting participation in physical activity for persons with SCI [10]. In Oslo, Norway, Sunnaas Rehabilitation Hospital Outpatient Clinique for Exercise (SRHOCE) was established in early 2015. The purpose was to offer exercise classes twice a week according to Canadian guidelines to persons with chronic SCI or other disabilities [5]. As far as we know, no extensive screening of health status in persons with SCI who exercise regularly according to these guidelines has been reported. The overall aim of this study was to make a descriptive examination of health status in persons with paraplegia (PP) and tetraplegia (TP) who exercise regularly at SRHOCE. The following research questions were formulated:

1. What is the physical health status regarding body composition, bone mineral density (BMD), lung function, and cardiorespiratory fitness for persons with PP and TP who exercise regularly at least twice a week?
2. What is their self-perceived QOL?

## Methods

### Study design

A non-controlled cross-sectional study, involving clinical examinations and a self-reported questionnaire, was performed during the autumn of 2016. In order to detect differences in cardiorespiratory fitness from previous examinations, a retrospective inspection of medical records was done. The study was conducted in cooperation between Sunnaas Rehabilitation Hospital (SRH) and the Norwegian School of Sport Sciences (NSSS).

### Training program

The training program at SRHOCE was developed in accordance with the Canadian guidelines, with aerobic activity and strength training two times a week. One class was chairobics, including strength exercises with manuals. The other class was circuit training, with aerobic exercises like boxing, interval wheeling, armergometer, and rope-slam. Strength exercises were performed with elastic bands, manuals, and pulley. Each class lasted 55 min in total, where at least 20 min consisted of aerobic activity. Strength training was performed with 8–10 repetitions in series of 3, with an intensity equivalent to 70–80% of 1 repetition maximum, as suggested [5]. Intensity level of the aerobic activity was pursued to be of a moderate to vigorous intensity, meaning an intensity between 60% and 80% of maximum heart rate (HR), as suggested [5]. The overall training program was carried out from January to June (summer holidays in July) and from August until middle of December each year.

### Subjects

Participants were recruited from SRHOCE. In total 18 participants, nine males and nine females, aged 41‒72 years with PP AIS [ASIA (American Spinal Injury Associations) Impairment Scale] A–B (*n* = 9), PP AIS C–D (*n* = 4), TP AIS A–B (*n* = 3), and TP AIS C (*n* = 2) signed up for this study. Inclusion criteria were persons with SCI who had followed the program for at least 6 months and had been exercising a minimum of two times a week for the last four weeks. All participants underwent a medical examination prior to training. No serious injuries were reported, and participants were declared fit to follow the training program.

### Data collection and measurements

All data were collected between September and November 2016. Questionnaires were delivered to participants and then returned after a workout session. Injury-related data and previous results from maximal exercise tests were extracted from the medical records at SRH. Clinical examination was done in the laboratories at NSSS and at the Division of Clinical Nutrition at Oslo University.

#### Body composition and bone measurement

Total body BMD and body composition were measured by dual energy X-ray absorptiometry (DXA) (Lunar iDXA Prodigy, Madison, USA). Soft tissue body composition, including fat mass (FM kg), percentage fat mass (FM %), lean tissue mass (LM kg), percentage lean mass (LM %), and visceral adipose tissue (VAT) were derived from the total body scan. Scanning was performed by two different operators who followed the standard procedures. The BMD *Z*-score was obtained from the manufacturer’s database by comparison with age- and gender-matched references. *Z*-score values <−2.0 were considered to be below the expected range for age according to The International Society for Clinical Densitometry (ISCD) [11].

#### Pulmonary function, ventilatory capacity, diffusion capacity, and airway inflammation

Pulmonary function, ventilatory capacity, and diffusion capacity were performed by using Masterscreen PTF (Jaeger Würzburg, Germany), according to European standards [12]. Pulmonary function measurements included maximal expiratory flow volume loops with forced vital capacity (FVC), forced expiratory volume in 1 s (FEV_1_), and maximal voluntary ventilation (MVV). Diffusion capacity of carbon monoxide (DL_CO_) was assessed with the single-breath method according to the standard protocol [13]. Results are expressed as percentage of predicted values based on age, sex, weight, and height [14]. The fraction of exhaled nitric oxide (FE_NO_) was assessed with the single-breath online test with a chemiluminescent analyzer EcoMedics CLD 88 Exhalyzer® (Eco Medics AG, Duerten, Switzerland). FE_NO_ is a marker of eosinophilic airway inflammation and was determined according to the standard protocol [15].

#### Cardiorespiratory fitness

Maximal oxygen uptake (VO_2max_) was measured with an incremental cardiopulmonary exercise test (CPET) on an arm ergometer (Ergometrics 800, Ergoline, Germany) until the participant reached exhaustion. The participant was connected to a gas analyzer (OxyconPro, Jaeger, Würtzburg, Germany) with a facemask covering the nose and the mouth. For safety reasons, blood pressure was assessed with an automatic blood pressure device (Welch Allyn Spot Vital Signs LXi, NY, USA). HR was determined with a heart rate monitor (Polar Electro Oy, Kempele, Finland). After warming up, exercise periods of 3 min were started with a minimum of 60 revolutions per minute (rpm). In a one-minute break between the exercise sessions, blood pressure, HR, and Borg Scale values were obtained. The start load and the increasing workload (from 5 to 30 W) for each exercise session were determined individually according to age, level of injury, and expected fitness status. The sessions continued until the participant reached exhaustion or were discontinued if the participants wanted to stop at any time. The end criteria for approved measurements consisted of several parameters: the Borg Scale ≥17, VO_2max_ reached a plateau even if the intensity increased, or the respiratory exchange ratio (RER) was >1.1. VO_2max_ was expressed as ml min^−1^ and ml kg^−1^ min^−1^, and minute ventilation (VE), HR, and RER were noted and used for further analysis. Breathing reserve (BR) was calculated (MVV-VE) and expressed as a percentage. The evaluation of the maximal HR was based on expected age-predicted HR_max_ from the HUNT fitness study [16]. The level of injury was corrected for when evaluated.

#### Quality of life

A Norwegian version of a questionnaire from The International SCI QOL Basic Data Set was used to assess QOL. This element was included in a questionnaire that was distributed to participants and then returned after the workout. The International SCI QOL contains three items that are each self-rated on a 0‒10 scale, with 0 = completely dissatisfied and 10 = completely satisfied [17]. The first item concerned satisfaction with personal circumstances, the second was satisfaction with physical health, and the third item concerned satisfaction with mental health. The time frame of self-rated satisfaction was four weeks.

### Statistical analysis

Statistical analysis was conducted with SPSS (Version 21, IBM SPSS Statistics Data Editor). Descriptive statistics are used to present basic characteristics and measurement outcomes. Due to the small sample size (*n* = 18), all data are presented in median and range (minimum, maximum) unless otherwise stated. To detect differences in injury level and sexes, variables were assessed with the Mann–Whitney *U* test for independent samples. For comparison of VO_2max_ measured in the present study and previously measured VO_2max_, One-Sample Wilcoxon Signed-Rank Test was used. Correlation between variables was evaluated using Spearman’s correlation analysis. Statistical significance was set at *p* ≤ 0.05. All tables and figures were compiled in Excel for Mac 2015 (version 15.29.1).

## Results

Descriptive characteristics are presented in Table 1. Except for height, none of the variables showed significant differences between males and females.

**Table 1:** Basic characteristics

Variable	Paraplegia (*n* = 13)	Tetraplegia (*n* = 5)	*p* value
Male			
Female			
Age (years)	58 (50–72)	47 (41–64)	0.15
Height (cm)	167 (153−190)	178 (162–181)	0.18
Weight (kg)	66 (46–113)	75 (37–85)	0.46
BMI (kg m^−2^)	24 (18–39)	24 (14–48)	0.88
Time (years) since injury	18 (4–54)	30 (19–47)	0.24
AIS A–B	9 (69%)	3 (60%)	
AIS C–D	4 (31%)	2 (40%)	
Exercise			
1 time a week	0	1 (20%)	
2 times a week	7 (54%)	1 (20%)	
>3 times a week	6 (46%)	3 (60%)	
More physically active now than before	8 (73%)	7 (100%)	
Smoking	2 (15%)	0	
Education level			
<7 years primary school	1 (8%)	0	
7–10 years primary school	0	1 (6%)	
1–2 years high school	2 (15%)	0	
3 years high school	4 (31%)	1 (20%)	
College/university bachelor	5 (39%)	0	
College/university master	1 (8%)	3 (60%)	
Civil status			
Married/cohabitant	10 (77%)	3 (60%)	
Single	3 (23%)	2 (40%)	
Experience pain	9 (69%)	3 (60%)	

Age, height, weight, and time since injury are presented in median with maximum and minimum in parentheses; remaining variables are presented in numbers counted with percent in parentheses

*BMI* body mass index, *AIS* ASIA (American Spinal Injury Associations) Impairment Scale

### Physiological results

Physiological differences between levels of injury were only significant for FVC and FEV_1_ (% predicted, *p* < 0.05) in persons with PP (AIS A–B) and PP (AIS C–D). FEV_1_ (% predicted) was the only significant different variable when comparing PP and TP (*p* < 0.05). Physiological variables for gender revealed significant differences only in FEV_1_ (% predicted) and total LM (*p* > 0.05). Due to minor differences, gender was merged in the other analyses. Physiological results for PP (AIS A‒B) and PP (AIS C–D), TP (AIS A‒B) and TP (C) are presented in Table 2. Individual results for body composition and BMD, pulmonary function, ventilatory capacity, and DL_CO_ are presented in Table 3. CPET outcomes are shown in Table 4. None of the participants had BR <15%.

**Table 2:** Differences in body composition, bone mineral density (BMD), lung function, diffusion capacity of carbon monoxide (DL_CO_), exhaled nitric oxide (FE_NO_), and maximal oxygen uptake (VO_2max_) in paraplegics AIS A–B and AIS C–D, and tetraplegics AIS A–B and AIS C

Variable	Paraplegia AIS A–B (*n* = 9)	Paraplegia AIS C–D (*n* = 4)	*p* value
BMD (g cm^−2^)	0.942 (0.885–1.312)	1.087 (1.059–1.203)	0.217
VAT (cm^3^)	971 (584–2619)	1385 (296–3054)	0.440
FM (kg)	28.02 (13.53–31.81)	36.09 (20.03–50.99)	0.090
FM (% of total body mass)	40 (25–51)	44 (35–51)	0.395
LM (kg)	36.41 (27.73–51.84)	41.33 (37.79–59.28)	0.217
LM (% of total body mass)	58 (48–72)	55 (48–63)	0.537
LM arms (g)	5518 (3752–8539)	4555 (4175–8618)	0.440
FVC (% predicted)	92.8 (59.6–121.3)	123.9 (91.0–130.9)	0.045
FEV_1_ (% predicted)	88.2 (61.1–119.0)	109.8 (91.0–119.1)	0.045
MVV (% predicted)	87.4 (60.1–133.5)	111.9 (100.0–124.9)	0.165
DL_CO_ (%)	72.3 (55.8–103.5)	91.9 (89.1–97.7)	0.123
FE_NO_ (ppb)	20.9 (6.7–42.2)	24.5 (16.1–37.6)	0.497
VO_2max_ (ml min^−1^)	1053 (914–1441)	1270 (1066–1663)	0.165
VO_2max_ (ml kg^−1^ min^−1^)	17.06 (12.15–25.28)	14.43 (14.13–22.63)	0.355
Variable	Tetraplegia AIS A–B (*n* = 3)	Tetraplegia AIS C (*n* = 2)	*p* value
BMD (g cm^−2^)	1.106 (1.034–1.299)	1.117 (1.095–1.138)	1.000
VAT (cm^3^)	2570 (1401–3379)	442 (89–795)	0.083
FM (kg)	34.38 (23.52–38.78)	17.48 (8.88–26.07)	0.236
FM (% of total body mass)	46 (32–47)	32 (26–38)	0.248
LM (kg)	44.11 (39.74–49.26)	34.11 (25.69–42.53)	0.248
LM (% of total body mass)	52 (52–65)	65 (60–10)	0.236
LM arms (g)	6506 (4726–7117)	3356 (3055–3657)	0.083
FVC (% predicted)	83.9 (66.1–91.3)	61.7 (46.0–77.3)	0.248
FEV_1_ (% predicted)	81.6 (68.6–83.2)	62.3 (43.0–81.5)	0.248
MVV (% predicted)	86.2 (82.6–90.8)	83.1 (57.0–109.2)	1.000
DL_CO_ (%)	67.2 (66.1–75.93)	69.9 (56.0–83.9)	1.000
FE_NO_ (ppb)	22.0 (19.4–29.2)	54.4 (49.2–59.6)	0.083
VO_2max_ (ml min^−1^)	1078 (845–1331)	1062 (771–1354)	1.000
VO_2max_ (ml kg^−1^ min^−1^)	14.29 (9.91–17.33)	20.06 (19.1–21.01)	0.083

Results are presented as median with minimum and maximum in parentheses

*VAT* visceral fat tissue, *FM* fat mass, *LM* lean mass, *FVC* forced vital capacity, *FEV*_*1*_ forced expiratory volume in 1 s, *MVV* maximal voluntary ventilation, *ppb* parts per billion, *ns* not significant,

*p* ≤ 0.05 = significant different

**Table 3:** Individual results of body composition and bone mineral density (BMD), pulmonary function, ventilatory capacity, and diffusion capacity of carbon monoxide (DL_CO_) (*n* = 18)

Sex	LL	AISA	LM (kg)	LM (%)	FM (%)	VAT (cm^3^)	BMD (g cm^2^)	BMD *Z-*score	FVC (%)	FEV_1_ (%)	MVV (l min^-1^)	MVV (%)	DL_CO_ (%)
M	PP	A	43.4	66	31	849	1.176	0.4	100.3	88.2	126.7	93.3	68.5
M	PP	A	35.7	63	35	980	0.943	−1.6	59.6	61.1	82.5	66.9	99.8
M	TP	C	42.5	60	38	795	1.095	−0.9	77.3	81.5	153.9	109.2	83.7
M	PP	D	59.3	52	46	3054	1.203	−0.4	91.0	91.0	104.8	100.0	93.0
M	PP	A	41.1	72	25	721	0.935	−1.5	73.1	76.7	107.7	85.1	55.8
M	PP	A	51.8	63	35	2619	0.885	−3.1	94.5	92.1	134.7	103.1	72.3
M	TP	B	49.3	65	32	1401	1.299	0.7	91.3	83.2	112.1	82.6	67.2
M	TP^a^	A	39.7	52	46	2570	1.034	0.1	83.9	81.6	113.2	90.8	75.9
M	TP	A	44.1	52	47	3379	1.106	−1.3	66.1	68.6	86.2	66.8	66.1
F	PP	A	30.4	48	51	971	0.925	−0.9	73.9	88.2	58.0	60.1	59.9
F	TP	C	25.7	70	26	89	1.138	2.0	46.0	43.0	56.5	57.0	56.0
F	PP	A	36.4	53	45	868	1.093	0.4	92.8	89.2	85.6	87.4	76.5
F	PP	A	41.8	56	43	1268	0.887	−1.7	88.1	86.3	84.9	84.5	68.6
F	PP	A	34.3	52	46	1090	1.312	2.8	107.0	96.7	133.0	133.5	78.3
F	PP	D	37.8	63	35	296	1.059	0.9	130.9	118.3	112.9	120.3	97.7
F	PP	C	40.0	48	51	1687	1.090	0.3	126.2	119.1	111.1	124.9	90.8
F	PP	D	42.6	57	42	1083	1.085	0.9	121.5	101.2	89.3	103.5	89.1
F	PP	A	27.7	58	41	584	0.942	−0.1	121.3	119.0	117.0	133.3	103.4

*M* male, *F* female, *LL* level of lesion, *PP* paraplegia, *TP* tetraplegia, *LM* lean mass, *LM*% % of total body fat, *FM*% % fat of total body mass, *VAT* visceral adipose tissue, *BMD* bone mineral density, *FVC* forced vital capacity, *FVC*% % of predicted values, *FEV*_1_ forced expiratory volume in 1 s, *FEV*_1_% % of predicted values, *MVV* maximal voluntary ventilation, *MVV*% % of predicted values, *DL*_*CO*_ diffusion capacity of carbon monoxide, *DL*_*CO*_% % of predicted values

^a^ Participant is originally a PP at level T1, but is considered as a TP in this study

**Table 4:** Individual results and outcomes of cardiopulmonary exercise test (*n* = 18)

Sex	LL	AISA	VO_2max_ ml min^−1^	VO_2max_ ml kg^−1^ min^−1^	RER_max_	VE_max_	HR_max_	Borg_max_	BR (%)
M	PP	A	1328	20.1	1.36	84	143	17	34
M	PP	A	1441	25.3	1.17	51	164		38
M	TP	C	1354	19.1	1.22	72	163	18	53
M	PP	D	1039	16.3	1.41	44	150		24
M	PP	A	1663	14.7	1.25	88	132		16
M	PP	A	969	17.1	1.14	60	139	17	44
M	TP	B	1249	15.2	1.26	79	135	17	41
M	TP^a^	A	1078	14.3	1.51	40	113		64
M	TP	A	1331	17.3	1.15	72	106	19	36
F	PP	A	845	9.9	1.02	41	106	18	52
F	TP	C	771	21.0	1.07	26	152		54
F	PP	A	991	14.5	1.41	51	166	19	40
F	PP	A	914	12.2	1.09	38	147	19	55
F	PP	A	1346	20.4	1.32	93	147	19	30
F	PP	D	1353	22.6	1.29	82	169	17	27
F	PP	C	1187	14.1	1.25	61	162	19	45
F	PP	D	1066	14.2	1.23	60	126	18	33
F	PP	A	1053	22.6	1.23	58	171		50

*M* male, *F* female, *LL* level of lesion, *PP* paraplegia, *TP* tetraplegia, *VO*_*2max*_ maximal oxygen uptake, *RER*_*max*_ maximal respiratory exchange ratio, *VE*_*max*_ maximal ventilation, *HR*_*max*_ maximal hart rate, *Borg*_*max*_ maximal Borg scale 6–20 (empty spaces caused missing data), *BR*% % breathing reserve

^a^ Participant is originally a PP at level T1, but is considered as a TP in this study

**Fig. 1:**
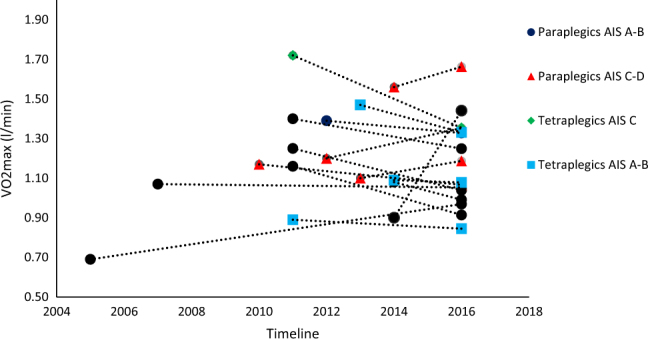
Dot plot of VO_2max_ (l min^−1^) from previous results and results from the present study in 2016. Time since previous test varied from 2 to 11 years. Each dot represents individual results from previous and recent VO_2max_ (l min^−1^). The dotted line binds together previous and present results and indicates a positive or negative direction (*n* = 16)

Previous measurements of VO_2max_ at SRH are sourced between years 2005 and 2014. The time since previous tests was 2‒11 years (median 4 years). Two of the participants had no previous test results and were therefore excluded from the analyses presented in Fig. 1. Figure 1 shows a dot plot of individual absolute VO_2max_ from the period before they started participating in the exercise group (median score VO_2max_ 1.17 l min^−1^) and from the present measurements (median score VO_2max_ 1.13 l min^−1^). The present measurements are reduced by 0.03 l min^−1^ when compared with previous measures. The difference was not significant (*p* > 0.05).

There was a significant negative correlation between VO_2max_ (ml kg^−1^ min^−1^) and total FM (Spearman’s rho = −0.7; *p* < 0.01). Similarly, a significant negative correlation was observed between VO_2max_ (ml kg^−1^ min^−1^) and total LM (Spearman’s rho = -0.57, *p* < 0.05). No correlation was observed between absolute or relative VO_2max_ and LM in participants’ arms.

### Self–reported quality of life

**Fig. 2:**
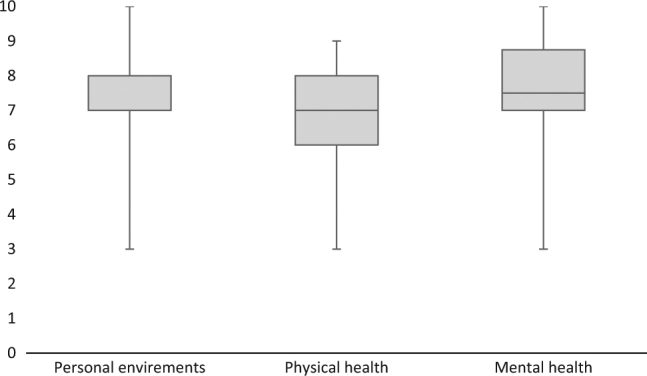
Self-reported satisfaction for personal circumstances, physical and mental health. The line in the middle of the box corresponds to the median, while the top and bottom of the boxes are the 25th and 75th percentiles. The whiskers show minimum and maximum range. Box plot for personal environments reveals that the 25th percentile is the same as the median

**Fig. 3:**
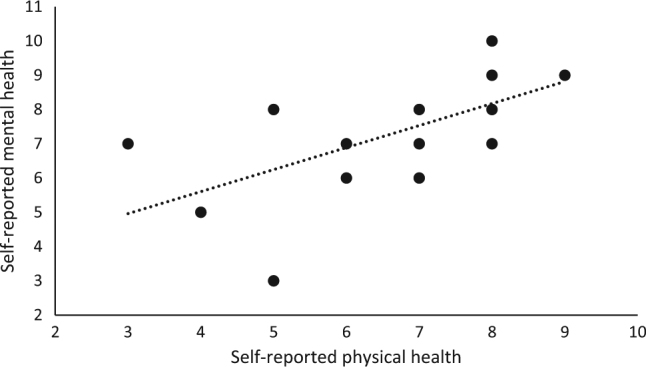
Correlation of self-reported physical and mental health. The correlation is significant positive (Spearman's rho = 0.739, *p* < 0.05)

Outcomes from the QOL questionnaires are presented in Fig. 2. Satisfaction with mental health was slightly higher (median 7.5) than satisfaction with personal environments and physical health (median 7). A positive correlation was seen between physical and mental health (Fig. 3).

## Discussion

The main findings of the present study were that persons with SCI who exercise regularly at SRHOCE had average to excellent cardiorespiratory fitness compared to an untrained SCI population [18]. This group also reported rather high QOL. Participants with TP had significantly reduced FEV_1_ (% predicted) compared with PP, but their BR revealed no ventilatory limitations. Despite variations in the groups, being slightly overweight was common for both groups.

### Body composition

In agreement with Wahman et al., overweight seemed to be a common variable when adjusting for specific body mass index (BMI) cutoff values for persons with SCI (median BMI = 24 kg m^−2^) [19]. A healthy BMI is proposed to be no more than 22/23 kg m^−2^ for persons with SCI, because each BMI grade consists of greater fat mass [20]. According to Kocina et al. [21], persons with SCI and total fat mass >25% (males) and 32% (females) are at high risk of developing secondary diseases. As Table 2 indicates, fat percent is above these percentages in both PP and TP. As a result of changes in body composition after a SCI, VAT is expected to be greater compared to the general population. An increase of 20% in VAT volume for every unit increase of BMI has been suggested [22]. This is consistent with our findings (median VAT = 1032 cm^3^), but the range in the present study revealed a huge variation (89‒3379 cm^3^).

### Bone mineral density

No difference was observed in total BMD between the PP and TP group, or between motor complete and incomplete injuries. This might be explained by the overall small sample size. However, we did not measure BMD in femur specifically and thus we cannot exclude osteoporosis in any of the groups. Our results of total body BMD are therefore of limited utility. Total BMD does not indicate a risk of osteoporosis in the femur, which is the limb at highest risk of developing osteoporosis in persons with SCI [23]. Total BMD revealed a risk of osteoporosis in three of the participants (BMD *Z*-score ≤ −2.0).

### Pulmonary function

We found significant differences in FEV_1_ (% predicted) due to injury level. In agreement with Linn et al. [24], reduction in pulmonary function was seen in persons with TP. MVV was within normal range in both groups [14], and DL_CO_ (% predicted) was slightly reduced in both groups. Because of the small sample size and unequal distribution of participants with TP (*n* = 5) versus PP (*n* = 13), we could not expect to detect significant differences. Although only FEV_1_ (% predicted) revealed significant difference between PP and TP, FVC (% predicted) showed borderline significant differences (*p* = 0.056). We can assume that with a greater number of participants in both groups, we might be able to detect a significant difference. Nonetheless, our findings indicate that pulmonary function is affected by higher SCI, indicating our results are within normal range for SCI and injury level. However, ventilation seemed not to be a limiting factor for cardiorespiratory fitness in the present study. In the general population, BR is normally between 20% and 40%, and values <15% may indicate ventilatory restrictions [25]. In the present study, BR varied from 16% to 64%, suggesting that pulmonary function did not restrict maximal exercise performance. This is presumably due to the involvement of small muscle groups with less oxygen demand when compared to a whole body workout.

### Cardiorespiratory fitness

We did not detect significant differences in VO_2max_ between PP and TT, or between motor compete and incomplete, which would have been expected based on previous research [18]. Simmons et al. [18] developed normative values with reference ranges for untrained males and females with SCI. Cardiorespiratory fitness values were grouped into reference categories of percentile ranking (i.e., poor <20%; fair 20–40%; average 40–60%; good 60–80%; excellent 80–100%) according to Janssen et al. [26]. Because of a small number of females (*n* = 26), Simmons et al. [18] did not define specific categories for females, other than provide female median values separately. In the present study, we could not show significant differences in VO_2max_ between the sexes, and the median from both sexes as a single group was used to categorize persons with TP and PP. The present study participants were significantly older (median age = 58 years in PP, 47 years in TP) than participants from the Simmons et al. study (mean age = 36 years in PP, 35 years in TP); thus, all of these categories will probably underestimate cardiorespiratory fitness in our sample. VO_2max_ is expected to decrease by about 10% every decade when a person is >30 years, and females tend to have 10% lower values than males [27]. When adjusting for age, categories for PP and TP in our sample were as follows: PP: VO_2max_ (ml kg^−1^ min^−1^) = good, PP: VO_2max_ (ml min^−1^) = good, TP: VO_2max_ (ml kg^-1^ min^-1^) = excellent, TP: VO_2max_ (ml min^-1^) = excellent. Thus, cardiorespiratory fitness in our sample can be described as good to excellent compared to an untrained SCI population.

A retrospective glance (Fig. 1) indicated that the cardiorespiratory fitness median was not significantly reduced compared with previous results. As we had no overview of any dietary changes during these years, we chose to compare the absolute VO_2max_ value (ml min^−1^). Still, eventually dietary changes may have affected the results. A medical records search provided retrospective test results between 2005 and 2014. With a median of four years since the previous test, we expected a greater decrease in VO_2max_. More precisely, according to the median we expected a 4% reduction, and VO_2max_ should have been reduced by 0.05 l min^−1^ [27]. This is 0.02 l min^−1^ more than what our analyses revealed. Even though the median tended to be lower, the dot plot (Fig. 1) indicates that some of the participants had improvements in their VO_2max_ (l min^−1^). Our descriptive data show that 81% of the participants were more physically active after the exercise group was created in April 2015. It is tempting to assume that the absence of a significant reduction in VO_2max_ is associated with regular exercise. Still, such a comparison entails several methodological weaknesses and should be interpreted with caution.

### Self-reported quality of life

It has been reported that persons with SCI have lower life satisfaction than the general population [28]. In our study, QOL was ranked as rather satisfying (Fig. 2). This is slightly higher than Norwegian SCI results from 2016 [29]. Results from NorSCIR were collected from persons with newly acquired SCI that occurred during their hospital stay. Since QOL has been reported to increase in accordance with time since injury, these findings were in consistence with previous research [30]. Although there have been different findings due to QOL and long-standing SCI, the relationship between QOL and physical activity appears to be valid [2, 28, 31]. Our findings support this relationship. We also found that self-reported satisfaction with physical health strongly correlated with mental health (Fig. 3). However, it has been reported that exercise capacity is related to higher life satisfaction [32]. No association between VO_2max_ and QOL was found in the present study (*p* > 0.05). The absence of significance may be explained by the small sample size or the complexity of what defines one’s QOL. It will be interesting to explore the impact of the social environment and support from peers in a group setting on the state of participants QOL in future studies.

### Methodical discussion

What strengthens the methods was that all of the tests, except for the DXA scan, were conducted by the same staff. The staff received the same training and followed the same protocol for each test. All clinical measurements were performed according to the gold standard test methods, were previously validated, and were considered to give robust, objective data. Evaluation of the CPET was done with guidance from staff at the clinical physiological laboratory at SRH. Considering end criteria and expected HR based on age and level of injury, there was reason to assume that the participants roughly achieved a maximal loaded test.

There were also study limitations. First, our cross-sectional study design made us unable to explore the causality of regular exercise and health. There is a great possibility that our associations were predicted by uncontrolled confounding factors. As for the small sample size and variations considering age and level and completeness of injuries, our findings may not be adequate to make generalizations about the SCI population. The small number of persons with TP also made it difficult to compare outcomes between persons with PP. However, the SCI population is scarce, and the sample of 18 males and females may provide useful information. We hope that our descriptive results can act as a basis for comparison in future research.

DXA measurements gave us concrete information about body composition. What may have challenged the reliability was that the measurements were performed at two different places by different staff. Some participants were not informed that their bladder should have been emptied, which may have influenced the results due to body water concentration. Metal was not corrected for and may have influenced total BMD. An important limitation in this study is that only total body BMD was measured. Total BMD does not correlate with potential osteoporosis in the distal femur, which has previously been reported to be the most sensitive bone site for assessing bone loss by DXA [22]. Thus, for future studies, we recommend specific assessments of the distal femur. Hemoglobin levels were automatically inserted in DL_CO_ assessments and probably do not concur with individual values and may have been a source of error. Confounding factors such as different settings and test leaders limit our comparison of absolute VO_2max_ with previous results. Finally, data sourced from the questionnaires involve weaknesses regarding validity and reliability. The international SCI QOL data set has not yet been validated, and some of the articles that were discussed used different questionnaires in the QOL survey.

Even though the classes at SRHOCE were established with a purpose of fulfilling a minimum of the physical activity guidelines, intensity was reserved for individual efforts. As Jörgensen et al. presented regarding exercise intensity [4], we do not know if participants in the present study achieved moderate to vigorous intensity due to the class structure or individual efforts. To get an indication, we could have used an HR monitor or other activity monitoring devices to obtain better control with regard to intensity. Our inclusion criteria are rather vague in the sense that some participants exercised more than what was considered to be the minimum of two times a week. This limits our ability to discuss whether fulfilling minimum guidelines was associated with our findings of health status.

### Considerations of health status and requests for future research

Our research contains limitations that limits its generalizability and ability to draw conclusions. We will cautiously state that we have surveyed health status, knowing that health is a very complex phenomenon. However, standardized methods have provided outcomes that can be used for comparison in future research. Hence, we encourage researchers to establish new interventions or controls to see if our assumptions are reasonable. A newly published article by Nightingale et al. calls for an increase in the SCI guidelines exercise intensity for physical activity [9]. For now, our findings support positive health associations with participation in physical exercise as presented by Pelletier et al. [6]. On this basis, we will continue to recommend that institutions and persons with SCI promote and develop exercise classes as established by the Canadian guidelines.

In conclusion, persons with SCI who exercise regularly at least twice a week responded with positive associations for health outcomes. Persons with PP and TP were slightly overweight, cardiorespiratory fitness indicated good to excellent aerobic capacity, and QOL was ranked as rather satisfying. Future research is needed to validate or strengthen our findings.
